# Supplementary material for: Differential organization of tonic and chronic B cell antigen receptors in the plasma membrane
Source: Nat Commun. 2019 Feb 18;10:820. doi: 10.1038/s41467-019-08677-1 (PMC6379438; doi:10.1038/s41467-019-08677-1)
Supplement: Supplementary file 1 — Supplementary Information [file 41467_2019_8677_MOESM1_ESM.pdf]

Supplementary Information

Differential organization of tonic and chronic B cell antigen receptors in the plasma membrane

Maria Angela Gomes de Castro[1], Hanna Wildhagen[1], Shama Sograte-Idrissi[1,2], Christoffer Hitzing[3], Mascha Binder[4], Martin Trepel[4,5], Niklas Engels[3,*], Felipe Opazo[1,2,*]

[1]Institute of Neuro- and Sensory Physiology, University Medical Center Göttingen, Humboldtallee 23, 37073 Göttingen, Germany

[2]Center for Biostructural Imaging of Neurodegeneration (BIN), University of Göttingen Medical Center, von-Siebold-Straße 3a, 37075 Göttingen, Germany

[3]Institute of Cellular and Molecular Immunology, University Medical Center Göttingen, Humboldtallee 34, 37073 Göttingen, Germany

[4]Department of Oncology and Hematology, BMT with section Pneumology, University Medical Center Hamburg-Eppendorf, Martinistr. 52, 20246, Hamburg, Germany

[5]Department of Hematology and Oncology, Augsburg Medical Center, Stenglinstr. 2, 86156, Augsburg, Germany

[*]**Corresponding author**: fopazo@gwdg.de, nengels@gwdg.de

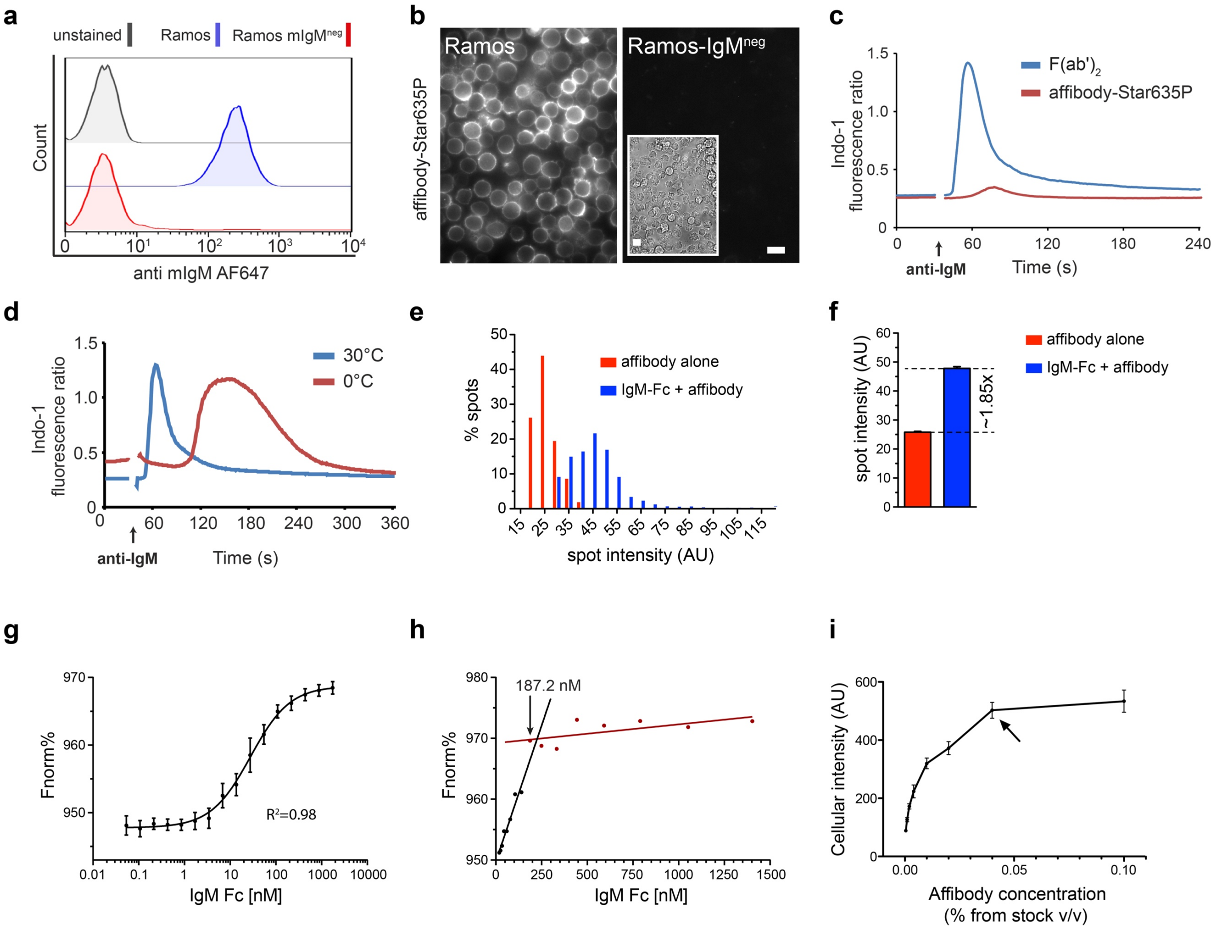

Supplementary Figure 1. Characterization of the anti-human IgM affibody conjugated to Star635P. a Surface staining of mIgM-BCRs in Ramos B cells expressing endogenous levels of mIgM (Ramos) and a subline lacking expression of mIgM (Ramos IgM^{neg}). Cells were stained with a polyclonal anti-IgM antibody and analyzed by flow cytometry. **b** Epifluorescence images of wild type and mIgM^{neg} Ramos B cells stained with affibody-Star635P. Fluorescent images were acquired under the same conditions and equally scaled to allow a direct comparison. Transmission light inset in the image on the right-hand side shows the presence of IgM^{neg} cells. Scale bars represent 10 µm. **c** Ca^{2+} mobilization analysis of wild type Ramos B cells stimulated (indicated by an arrow) with either polyclonal anti-IgM F(ab')$_2$ (blue curve) or the Star635P-conjugated monovalent anti-IgM affibody (red curve). **d** Ca^{2+} mobilization of Ramos B cells stimulated with polyclonal anti-IgM F(ab')$_2$ either at 30°C (blue curve) or in ice-cold buffer (0°C, red curve). **e** Fluorescence intensity distribution of Star635P-labelled affibody alone (red bars, ~1,000 spots) or pre-mixed with monomeric IgM-Fc (blue bars, ~4,000 spots) adsorbed on glass coverslips and imaged by STED microscopy. **f** Bar graph showing the mean fluorescence intensities of single spots. Error bars represent the 95% confidence interval. **g** Thermophoresis affinity determination of the Star635P-labelled affibody to recombinant IgM-Fc (estimated Kd: 27.8 nM). R^2 indicates the accuracy of the fit. Error bars represent the standard deviation of five independent experiments. **h** Binding stoichiometry determined using thermophoresis. A 400 nM solution of affibody-Star635P was mixed with increasing concentrations of IgM-Fc. Linear regressions of saturated and unsaturated points are displayed. Thermophoresis signals become constant at ~187.2 nM of IgM-Fc, suggesting saturation of binding at this point and therefore a ~2.1:1 binding stoichiometry (affibody:IgM-Fc). **i** Wild

type Ramos B cells were stained with increasing concentrations of affibody-Star635P to determine the optimal concentration needed for saturated labeling of mIgM-BCRs on the cell surface. Dilutions ranged from 1:10 (0.1%) to 1:1000 (0.001%) from our stock of fluorescently-labeled affibody. After background correction, regions of interest containing whole cells were manually selected and the average intensity of whole cells was calculated. Values represent the average of cellular fluorescence intensities from >50 cells per concentration point. Error bars represent the standard error of the mean. The arrow indicates the final concentration that was used throughout the manuscript.

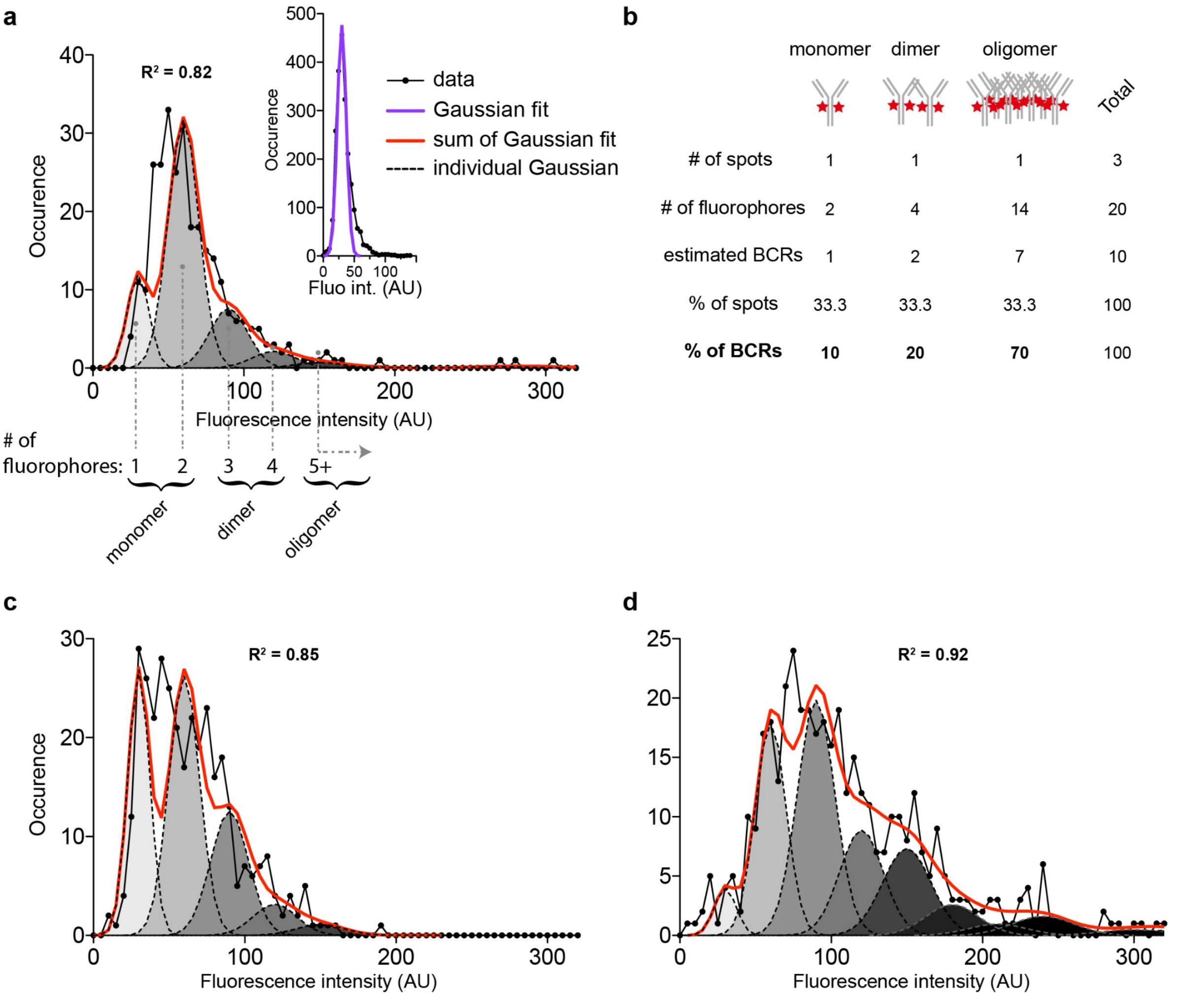

Supplementary Figure 2. Gaussian fits of BCR spot intensity distributions. a Histograms showing the spot fluorescence intensity distribution (black solid line) of all Ramos B cell membranes, a fit to the data with the model being a sum of Gaussian distributions as described in the Methods (red trace, R^2 indicates the accuracy of the fit) and the individual Gaussian distributions (grey traces, dotted lines) as obtained from the fit. The peak position of the single affibody-Star635P intensity distribution (inset) was obtained by a single Gaussian fit (purple trace, here 30 AU) and was used as reference for the BCR data, i.e. the position of each individual Gaussian curve (gray traces) was forced to be a multiple of this value. From the area under each individual Gaussian curve we determined the proportions of spots on the membrane sheets that contained 1, 2, 3, 4 or ≥5 fluorophores. **b** Hypothetical example of how the numbers of BCR units per spot were calculated. Spots displaying the intensity of 2, 4 or 14 Star635P fluorophores correspond to either monomeric, dimeric or oligomeric (≥3) mIgM-BCRs, respectively. In this example with 3 spots, the spot analysis would reveal that 33.3% **of the spots** represent monomeric mIgM, 33.3% represent dimers and another 33.3% represent mIgM oligomers. However, an analysis at the BCR level would reveal that 10% **of BCRs** are monomers, 20% of the BCRs are organized as dimers and 70% exist as oligomers. The % of BCRs obtained are the numbers (% of mIgM-BCRs) used for Fig. 1d, g, j and Fig 3c, f, i. **c** and **d** correspond to the raw fluorescence distribution of all membranes analyzed from primary B cells (Fig. 1g) and Ramos B cells + anti-IgM F(ab')$_2$ fragments (Fig. 1j) respectively. Histogram´s source data are provided as a Source Data file.

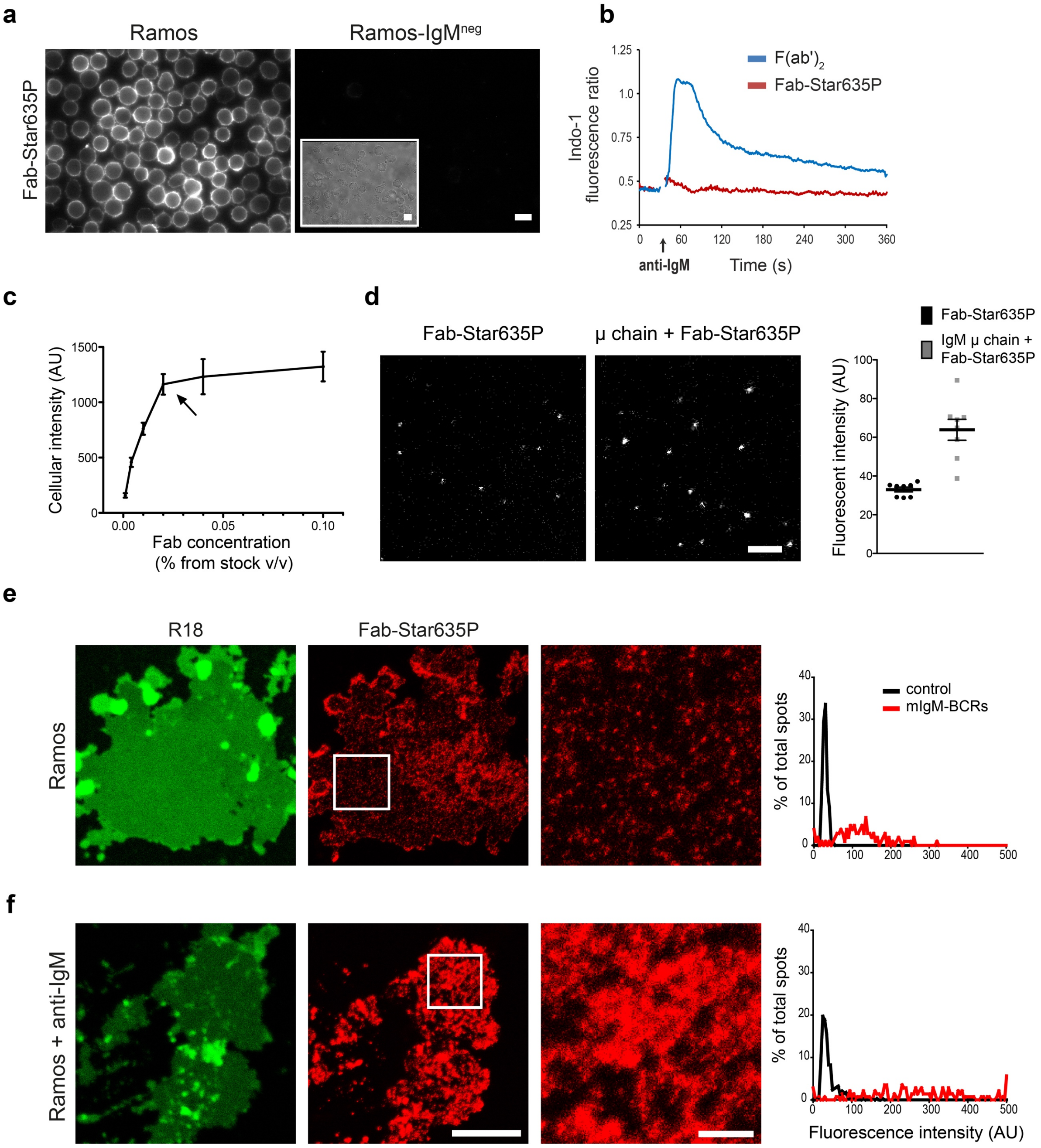

Supplementary Figure 3. Characterization of a Star635P-conjugated polyclonal anti-human IgM Fab. a Epifluorescence images of wild type and IgM-negative (IgM^{neg}) Ramos B cells stained with a monovalent Star635P-conjugated Fab against human IgM (Fab-Star635P). Fluorescent images were acquired under the same conditions and equally scaled to allow a direct comparison. Transmission light inset in the image on the right-hand side shows the presence of IgM^{neg} cells. Scale bars represent 10 µm. **b** Ca^{2+} mobilization analysis of wild type Ramos B cells stimulated (indicated by an arrow) with either polyclonal anti-IgM F(ab')$_2$ (blue curve) or the Star635P-conjugated monovalent anti-IgM Fab (red curve). **c** Wild type Ramos B cells were stained with increasing concentrations of Fab-Star635P to establish the optimal concentration needed for saturated labeling of mIgM-BCRs on the cell surface. Dilutions ranged from 1:10 (0.1%) to 1:1000 (0.001%) from our stock of fluorescently-labeled Fab. After background correction regions of interest containing whole cells were manually selected and the average intensity of whole cells was calculated. Values represent the average of cellular

fluorescence intensities from >50 cells per concentration point. Error bars represent the standard error of the mean. The arrow indicates the final concentration that was used in the experiments shown. **d** STED images of single Fab-Star635P and Fab-Star635P mixed with a monomeric Ig μ heavy chain seeded on glass coverslips (scale bar 1μm). The bar graph shows the mean fluorescence intensity of single spots and error bars represent the standard error of the mean from eight independent experiments. **e, f** Confocal images of plasma membrane sheets stained with R18 (pseudocolored in green) and STED images showing the Fab-Star635P fluorescence signal derived from either untreated Ramos B cells (**e**) or cells that were BCR-activated with a monoclonal anti-IgM antibody (**f**). Membrane sheets were analyzed as described for Figure 1. Example histograms showing the fluorescence intensity distributions of Fab-Star635P-conjugated on coverslips (black curves, control) and mIgM-spots present in membrane sheets (red curves, mIgM-BCRs).

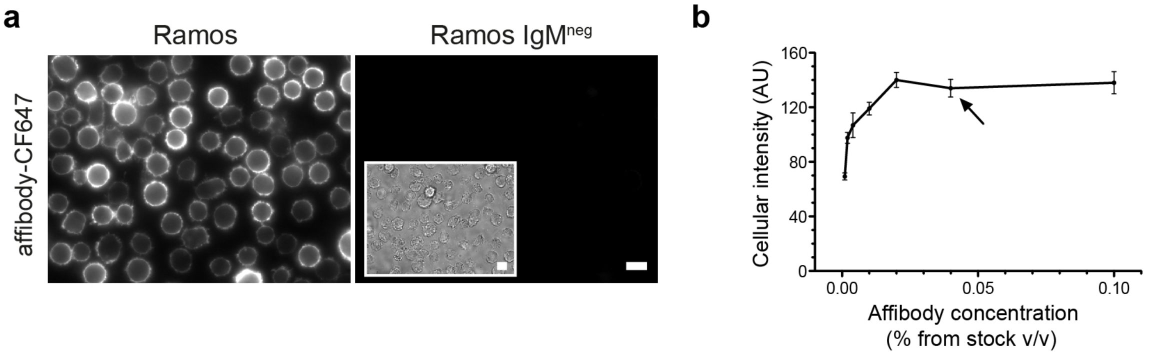

Supplementary Figure 4. Characterization of the anti-human IgM affibody conjugated to CF^{TM}647. a Epifluorescence images of wild type and IgM-negative (IgM^{neg}) Ramos B cells were obtained following staining with affibody-CF^{TM}647. Fluorescent images were acquired under the same conditions and equally scaled to allow for direct comparison. Transmission light inset in the image on the right-hand side shows the presence of cells. Scale bars represent 10 µm. **b** Wild type Ramos B cells were stained with increasing concentrations of affibody-CF^{TM}647 to establish the optimal concentration needed for saturated labeling of mIgM-BCRs on the cell surface. Dilutions ranged from 1:10 (0.1%) to 1:1000 (0.001%) from our stock of CF^{TM}647-labeled affibody. After background correction regions of interest containing whole cells were manually selected and the average intensity of whole cells was calculated. Values represent the average of cellular fluorescence intensities from >50 cells imaged per concentration point. Error bars represent the standard error of the mean. The arrow indicates the final concentration that was used throughout the manuscript.

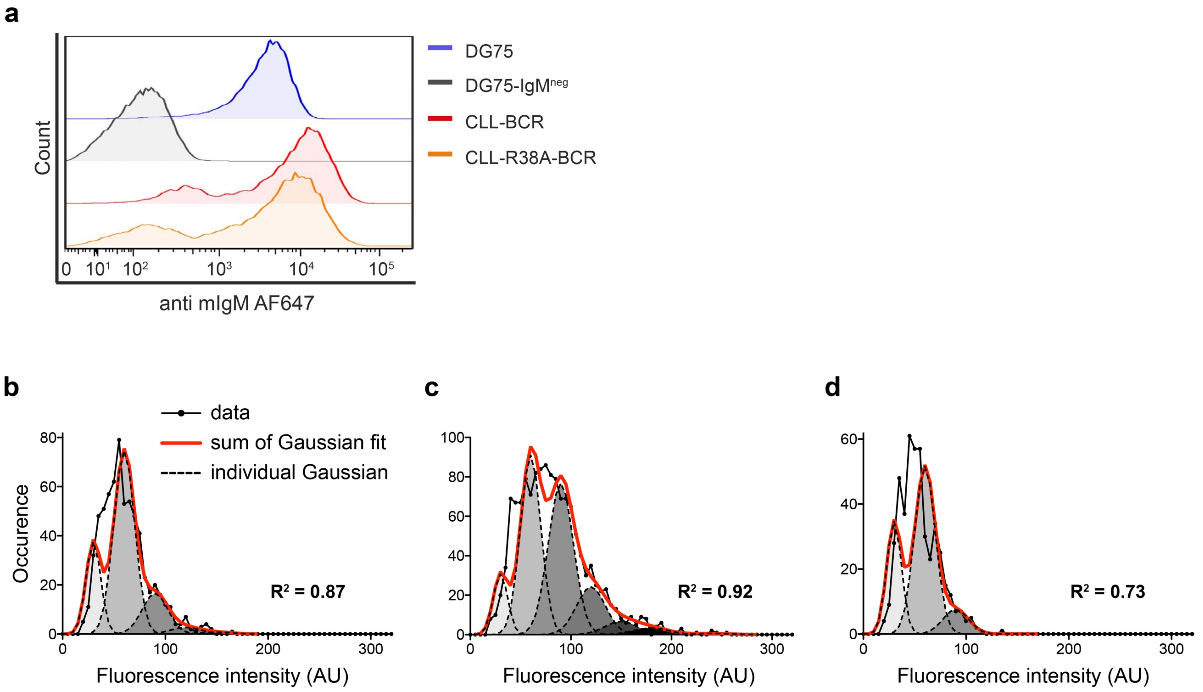

Supplementary Figure 5. Flow cytometric analysis of tonic and chronic surface mIgM-BCR variants in DG75 B cells. a Surface expression of different mIgM-BCR variants was analyzed by staining with a Cy5-labeled polyclonal anti-human IgM antibody. Surface expression of the endogenous BCR of DG75 cells is shown in blue, the dark grey curve shows DG75 cells that were sorted for mIgM negativity (IgM^{neg}). The red curve shows IgM-negative DG75 B cells in which the expression of a CLL-derived mIgM was induced by addition of doxycyline for 18 hours. Expression of the CLL-R38A-BCR -mutant is shown in orange. **b, c and d** show fluorescence intensity distributions as in Supplementary Fig. 2 of all membranes analyzed from DG75 (**b**), DG75 expressing the CLL-BCR (**c**) and the CLL-R38A-BCR mutant (**d**) displayed in Fig. 3. R^2 indicates the accuracy of the fit. Histogram´s source data are provided as a Source Data file.

Supplementary Table 1

Primers

PCR primers for amplification of the human membrane-bound immunoglobulin µ heavy chain constant part cDNA	
Name	Sequence (5' → 3')
hµmBNX-fwd	CCGGATCCGCGGCCGCTATGGTCTCGAGCGGGAGTGCATCCGCCCCAACC
hµmBam-rev	TCCGGATCCTCATTTCACCTTGAACAAGGTGAC
Primers for site-directed mutagenesis to introduce an arginine-to-alanine amino acid exchange at position 38 in CLL #025 heavy chain V region cDNA	
Name	Sequence (5' → 3')
HC025_R38A-fwd	GCTATCAGCTGGGTGGCACAGGCCCCTGGAC
HC025_R38A-rev	GTCCAGGGGCCTGTGCCACCCAGCTGATAGC
