# Supplementary material for: Differential organization of tonic and chronic B cell antigen receptors in the plasma membrane
Source: Nat Commun. 2019 Feb 18;10:820. doi: 10.1038/s41467-019-08677-1 (PMC6379438; doi:10.1038/s41467-019-08677-1)
Supplement: Supplementary file 3 — Reporting Summary [file 41467_2019_8677_MOESM3_ESM.pdf]

nature research

Corresponding author(s): Felipe Opazo, Niklas Engels

Last updated by author(s): Jan 17, 2019

Reporting Summary

Nature Research wishes to improve the reproducibility of the work that we publish. This form provides structure for consistency and transparency in reporting. For further information on Nature Research policies, see Authors & Referees and the Editorial Policy Checklist.

Statistics

For all statistical analyses, confirm that the following items are present in the figure legend, table legend, main text, or Methods section.

n/a	Confirmed	
☐	☒	The exact sample size (n) for each experimental group/condition, given as a discrete number and unit of measurement
☐	☒	A statement on whether measurements were taken from distinct samples or whether the same sample was measured repeatedly
☒	☐	The statistical test(s) used AND whether they are one- or two-sided<br>*Only common tests should be described solely by name; describe more complex techniques in the Methods section.*
☒	☐	A description of all covariates tested
☒	☐	A description of any assumptions or corrections, such as tests of normality and adjustment for multiple comparisons
☐	☒	A full description of the statistical parameters including central tendency (e.g. means) or other basic estimates (e.g. regression coefficient) AND variation (e.g. standard deviation) or associated estimates of uncertainty (e.g. confidence intervals)
☒	☐	For null hypothesis testing, the test statistic (e.g. F, t, r) with confidence intervals, effect sizes, degrees of freedom and P value noted<br>*Give P values as exact values whenever suitable.*
☒	☐	For Bayesian analysis, information on the choice of priors and Markov chain Monte Carlo settings
☒	☐	For hierarchical and complex designs, identification of the appropriate level for tests and full reporting of outcomes
☒	☐	Estimates of effect sizes (e.g. Cohen's d, Pearson's r), indicating how they were calculated

Our web collection on statistics for biologists contains articles on many of the points above.

Software and code

Policy information about availability of computer code

Data collection	Flow cytometry: Data collection was done using FACS Diva software (BD Biosciences).<br>ÄKTA chromatography: UNICORN™ 7.0.2 software (GE Healthcare Life Sciences)<br>Binding affinity measurements, Microscale Thermoforesis - Monolith NT.115: M.O. Control v1.4.3 (NanoTemper Technologies GmbH)<br>STED and confocal images: Leica LAS AF v1.7.0<br>Epifluorescence microscopy: Olympus IX71 microscope equipped with a cellSens Software
Data analysis	Flow cytometry: Data analysis was done using FlowJo (FlowJo LLC) and Microsoft Excel (Microsoft).<br>Data analysis: SigmaPlot for Windows Version 10.0, Build 10.0.1.25, Copyright 2006 Systat Software Inc.<br>Data Analysys: Matlab Version 7.5.0.342 (R2007b), 2007, Mathworks Inc.<br>Image processing: ImageJ, Fiji, NIH, Schindelin, J.; Arganda-Carreras, I. & Frise, E. et al. (2012), "Fiji: an open-source platform for biological-image analysis", Nature methods 9(7): 676-682, PMID 22743772, doi:10.1038/nmeth.2019

For manuscripts utilizing custom algorithms or software that are central to the research but not yet described in published literature, software must be made available to editors/reviewers. We strongly encourage code deposition in a community repository (e.g. GitHub). See the Nature Research guidelines for submitting code & software for further information.

Data

Policy information about availability of data

All manuscripts must include a data availability statement. This statement should provide the following information, where applicable:

- Accession codes, unique identifiers, or web links for publicly available datasets
- A list of figures that have associated raw data
- A description of any restrictions on data availability

The source data underlying Figs 1d, g, j, and 3c, f, i, and Supplementary Figs 2 and 5b, c, d are provided as a Source Data file.

Field-specific reporting

Please select the one below that is the best fit for your research. If you are not sure, read the appropriate sections before making your selection.

☒ Life sciences ☐ Behavioural & social sciences ☐ Ecological, evolutionary & environmental sciences

For a reference copy of the document with all sections, see nature.com/documents/nr-reporting-summary-flat.pdf

Life sciences study design

All studies must disclose on these points even when the disclosure is negative.

Sample size	Sample size calculation were not performed a priori. The aim was to collect as many data as possible considering the difficulty of the experimental conditions. We made sure to have at least 3-4 fully independent experiments with several measurement in each (15-30). Sample size provided a sufficient accuracy to distinguish statistically significant differences between negative and positive control.
Data exclusions	In principle no data were excluded from the analysis. However, while analyzing images, some were not processed further due to a lack of proper lens focus.
Replication	We made sure to have at least 3-4 fully independent experiments with several measurement of individual cells in each experiment (15-30). Standard deviations of the independent experiments were not very large, confirming that we were able to reproduce our findings with an acceptable experimental error.
Randomization	There was no need to systematically randomize our samples. However, individual samples were stained and imaged without a specific order.
Blinding	Blinding was not necessary in our experimental setup. The same person preparing the samples was acquiring the microscopy data.

Reporting for specific materials, systems and methods

We require information from authors about some types of materials, experimental systems and methods used in many studies. Here, indicate whether each material, system or method listed is relevant to your study. If you are not sure if a list item applies to your research, read the appropriate section before selecting a response.

Materials & experimental systems

n/a	Involved in the study	
☐	☒	Antibodies
☐	☒	Eukaryotic cell lines
☒	☐	Palaeontology
☒	☐	Animals and other organisms
☐	☒	Human research participants
☒	☐	Clinical data

Methods

n/a	Involved in the study	
☒	☐	ChIP-seq
☐	☒	Flow cytometry
☒	☐	MRI-based neuroimaging

Antibodies

Antibodies used	Polyclonal goat anti-human IgM-AF647 (Southern Biotech, cat. #2020-31).<br>Anti-IgM affibody® ab36088 (Abcam, Cambridge, UK).<br>AffiniPure F(ab')$_2$ Fragment Goat Anti-Human IgM, Fc5μ fragment specific (Jackson ImmunoResearch, West Grove, PA, USA, cat. #109-006-129).
Validation	Polyclonal goat anti-human IgM-AF647 (Southern Biotech, cat. #2020-31): The antibody was validated for FACS applications by the supplier (Southern Biotech, Birmingham, AL, USA). The specificity for human IgM was also confirmed by our own experiments shown in Supplementary Figures 1a and 5a.<br>Anti-IgM affibody® ab36088 (Abcam, Cambridge, UK): validated by quantitative ELISA by the supplier (Abcam, Cambridge, UK) and by our own experiments in Supplementary Figures 1b and 4a.

AffiniPure F(ab')$_2$ Fragment Goat Anti-Human IgM, Fc5µ fragment specific (Jackson Immuno Research, UK): the Fab specificity was validated for immunoelectrophoresis and/or ELISA. References published in the internet site from the supplier: PLOS ONE DOI:10.1371/journal.pone.0158641 July 5, 2016; NATURE COMMUNICATIONS | 7:11138 | DOI: 10.1038/ncomms11138)

Eukaryotic cell lines

Policy information about cell lines

Cell line source(s)	The human Burkitt lymphoma cell lines DG75 and Ramos were purchased from the German Collection for Microorganisms and Cell Cultures (DSMZ), Braunschweig, Germany.
Authentication	Authentication of cell lines was done by the DSMZ cell bank. So far neither DG75 nor Ramos were reported by the ICLAC to be misidentified.
Mycoplasma contamination	All cells were negative for mycoplasma in routine PCR assays.
Commonly misidentified lines (See ICLAC register)	*Name any commonly misidentified cell lines used in the study and provide a rationale for their use.*

Human research participants

Policy information about studies involving human research participants

Population characteristics	Human research participants were two healthy males (age: 39 & 42 years, ethnicity: caucasian).
Recruitment	Two human research participants volunteered as blood donors. No specific selection criteria were applied other than health status and good physical condition on the day of the experiment.
Ethics oversight	Experiments involving human participants were approved by the ethical review committee of the University Medical Center Göttingen (case number 11/6/17) and were performed in accordance with relevant guidelines and regulations. An informed consent was obtained from the participants.

Note that full information on the approval of the study protocol must also be provided in the manuscript.

Flow Cytometry

Plots

Confirm that:

- [x] The axis labels state the marker and fluorochrome used (e.g. CD4-FITC).
- [x] The axis scales are clearly visible. Include numbers along axes only for bottom left plot of group (a 'group' is an analysis of identical markers).
- [ ] All plots are contour plots with outliers or pseudocolor plots.
- [ ] A numerical value for number of cells or percentage (with statistics) is provided.

Methodology

Sample preparation	Cell lines were harvested from culture dishes by centrifugation, washed in phosphate-buffered saline (PBS) and stained with either the indicated antibodies or loaded with Indo1-AM (as indicated). After extensive washing, cells were used for flow cytometry measurements.
Instrument	Data acquisition was done using an LSRII flow cytometer (BD Biosciences).
Software	FACS data were collected using FACS Diva software and analyzed using FlowJo and Microsoft Excel (Ca2+ measurements).
Cell population abundance	n.a.
Gating strategy	In the analyses of flow cytometric data, cellular debris was excluded by applying a forward/side scatter gate on live cells. This is standard procedure in FACS experiments.

- [ ] Tick this box to confirm that a figure exemplifying the gating strategy is provided in the Supplementary Information.
